# Long noncoding RNA TUG1 promotes proliferation, migration and cisplatin resistance in oral squamous cell carcinoma: TUG1 promotes proliferation, migration and cisplatin resistance in OSCC

**DOI:** 10.3724/abbs.2023090

**Published:** 2023-05-25

**Authors:** Shuyan Liu, Weirong Wang, Lingyun Ye, Chanjuan Liu, Wei Xiao, Jinxing Gao

**Affiliations:** 1 Center for Plastic & Reconstructive Surgery Cancer Center Department of Dental Medicine Zhejiang Provincial People’s Hospital (Affiliated People’s Hospital Hangzhou Medical College) Hangzhou 310014 China; 2 School of Pharmacy Zhejiang University Hangzhou 310027 China

Oral squamous cell carcinoma (OSCC) is a malignant tumor with a high metastasis rate and high recurrence rate, accounting for approximately 90% of oral cancers. Worldwide, there are more than 350,000 new cases each year. Approximately 50% of patients with OSCC are in an advanced stage at the time of treatment. Although there has been great progress in the diagnosis and treatment of tumors in recent years, the prognosis of OSCC has not been significantly improved. Studies have suggested that long noncoding RNAs (lncRNAs) are involved in the progression of cancer and can be used as cancer markers [1].

LncRNAs are generally defined as RNA transcripts that are longer than 200 nucleotides and have no protein-coding potential. Approximately 4%‒9% of the sequences in mammalian genomes produce transcripts as lncRNAs, while mRNA with protein coding function is only 1%. LncRNA is a kind of noncoding RNA transcript and it can regulate the level of miRNA and downstream gene expression, playing an important role in the regulation of gene expression and tumor formation, proliferation, invasion and metastasis [2]. With the research in depth on the molecular mechanism of OSCC occurrence and development, it shows that lncRNAs in OSCC are not only closely related to the clinicopathological characteristics of patients, but also affect the biological behavior of tumor cells by regulating miRNA or signaling pathways. It plays an important role in promoting or inhibiting cancer growth [3]. LncRNAs can not only be used as tumor diagnostic and prognostic factors, but also as tumor markers and targets for tumor therapy. Therefore, the study of differentially expressed lncRNAs is of great significance for the early diagnosis and treatment of OSCC.

In recent years, studies have shown that lncRNA TUG1 is highly expressed in lung cancer by Gene Expression Omnibus (GEO) and The Cancer Genome Atlas (TCGA) analyses [4]. In ovarian cancer, pancreatic cancer and other tumors, TUG1 is also involved in the regulation of cell proliferation, apoptosis and the EMT signaling pathway [ 5, 6] . TUG1 possesses a 6.7-kb RNA sequence and regulates the cysteine derivative taurine, which is essential for neural development. TUG1 is highly expressed in many tumors, and the mechanism may be related to the formation of chromatin modification complexes and gene regulation. LncRNA TUG1 has been reported to activate the Wnt/β-catenin pathway. Over-expression of TUG1 induces doxorubicin (Dox) resistance in bladder cancer (BC) cell lines [7]. However, the knockout of TUG1 gene inhibited the resistance of BUC cells to Dox. It also demonstrated that TUG1 affects cell growth and drug-resistant SCLC. However, the expression and regulatory mechanism of TUG1 in OSCC are unclear. In this study, the expressions of TUG1 in OSCC tissues and cells were detected, the relationship between TUG1 expression and the prognosis of OSCC patients was analyzed, and the potential mechanism of TUG1 expression on the proliferation, migration, apoptosis and drug resistance of OSCC cells was explored.

To overexpress TUG1 protein, lentivirus vectors carrying *TUG1* and the green fluorescent protein gene or the *TUG1* gene alone were successfully constructed. TUG1 and green fluorescent protein were successfully expressed in human tongue squamous cell carcinoma cell line SCC9 (Hunan Fenghui Biotechnology Co., Ltd, Changsha, China), compared with cells infected with the lentivirus empty vectors which were used as the negative control (NC). Meanwhile, knockdown of *TUG1* was achieved by transfection of SCC9 cells with si-TUG1 (5′-GGUUGGUUGUGGGAUUUCUTT-3′; GenePharma, Shanghai, China), using si-NC (Cat No. A01001; GenePharma) as the negative control.

The optimal MOI was 40 at 72 h after infection with lentivirus carrying *TUG1* and green fluorescent proteins (data not shown). The level of TUG1 in the TUG1-OE group was significantly higher than that in the control (NC) group, and the level of TUG1 in the si-TUG1 group was significantly lower than that in the si-NC group ( Figure 1A). The transwell assay results showed that the number of migrated cells in the TUG1-OE group was higher than that in the control groups ( *P*<0.001), and the number of migrated cells in the si-TUG1 group was lower than that in the si-NC group ( *P*<0.001; Figure 1B). These results suggested that lncRNA TUG1 significantly promoted SCC9 cell migration *in vitro*. To further verify the role of lncRNA TUG1 in SCC9 cell apoptosis, the cell apoptosis rate was detected by Annexin V/PI staining and flow cytometry. The results showed that cell apoptosis was increased in the si-TUG1 group compared with that in the si-NC group, and cell apoptosis was decreased in the TUG1-OE group compared with that in the control group ( Figure 1C).

**Figure 1:**
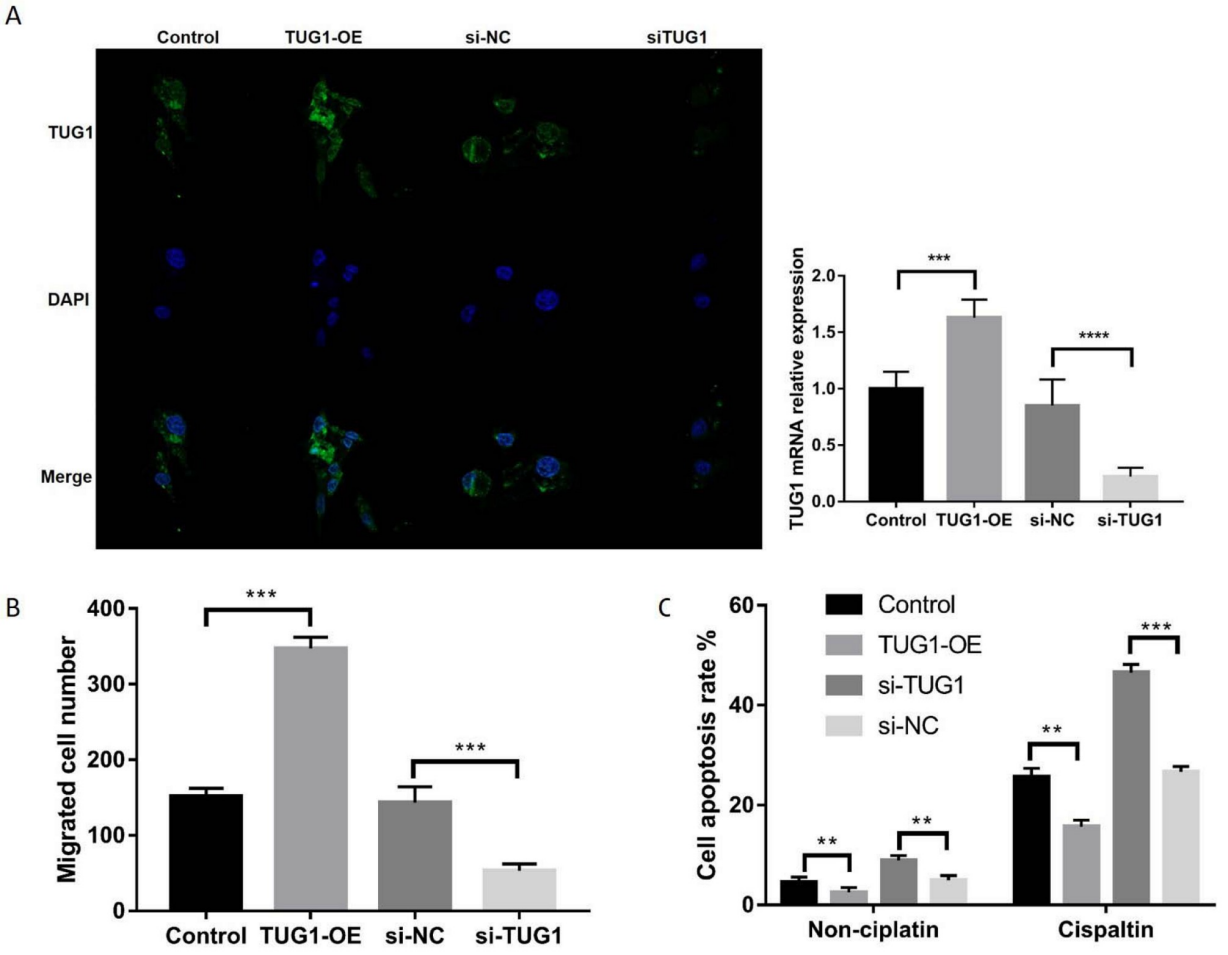
Figure 1 Migration and apoptosis of OSCC cells after overexpression or knockdown of *TUG1* (A) TUG1 protein expressions in OSCC cells of the TUG1 si-TUG1, si-NC, control (NC) and TUG1-OE groups at 72 h after infection detected by immunofluorescence staining. RT-PCR results showing TUG1 mRNA expressions in the si-TUG1, si-NC, control and TUG1-OE groups at 72 h after infection. (B) A transwell assay was performed to determine the migration of SCC9 cells infected with lentivirus carrying control, TUG1-OE, si-TUG1 and si-NC plasmids. The number of migrated cells was detected in control, TUG1-OE, si-NC and si-TUG1 groups. Data are presented as the mean±SD. ***P<0.001 indicates that the difference is extremely significant. **P<0.01 indicates that the difference is significant. (C) The apoptosis rates of OSCC cells in the control, TUG1-OE, si-TUG1, and si-NC groups were determined by flow cytometric analysis after treatment with cisplatin.

CKK‐8 assay was performed to explore the viability of SCC9 cells. The cell viability of SCC9 cells was inhibited after 12 h,24 h and 48 h of treatment with increasing concentrations of cisplatin. The death rate was <50% when the cells were treated with 4 μM cisplatin for 72 h ( Table 1). After treatment with cisplatin (4 μM) for 12 h,24 h and 48 h, cell viability was decreased, and apoptosis was increased with time. The cell viability of the TUG1-OE group was significantly higher than that of the NC and si-TUG1 groups ( *P*<0.001). The cell viability of the si-NC group was significantly higher than that of the si-TUG1 group ( *P*<0.001) and the cell viability gradually decreased within 48 h ( Figure 2A–C). The OD _450nm_ value of the TUG1-OE group was higher than that of the NC group after treatment with cisplatin for 12 h,24 h and 48 h. On the contrary, OD _450nm_ value in si-TUG1 group was lower than that in NC group after treatment with cisplatin for 12 h,24 h and 48 h ( Figure 2D). The apoptosis rate in the TUG1-OE group was lower than that in the NC group after treatment with cisplatin for 12 h,24 h and 48 h. In contrast, the apoptosis rate in the si-TUG1 group was higher than that in the si-NC group after treatment with cisplatin for 12 h,24 h and 48 h ( Figure 2E). Our transwell assay results also showed that cell migration of the TUG1-OE group was 1.5~2- *fold* higher than that of the control group. Cisplatin could obviously inhibit the proliferation of SCC9 cells and promote cell apoptosis. Knockdown of *TUG1* in cisplatin-treated SCC9 cells significantly inhibited cell growth, suggesting that TUG1 could promote drug resistance, migration and proliferation of SCC9 cells. TUG1 lncRNA acts as a sponge to adsorb downstream targets involved in drug resistance and downstream genes by changing the activity of transcription factors related to drug resistance genes. Although the results proved that TUG1 regulated OSCC drug resistance, the mechanism is still unclear. The molecular mechanism by which lncRNA TUG1 promotes the drug resistance of OSCC cells still needs to be explored in further studies.

**Figure 2:**
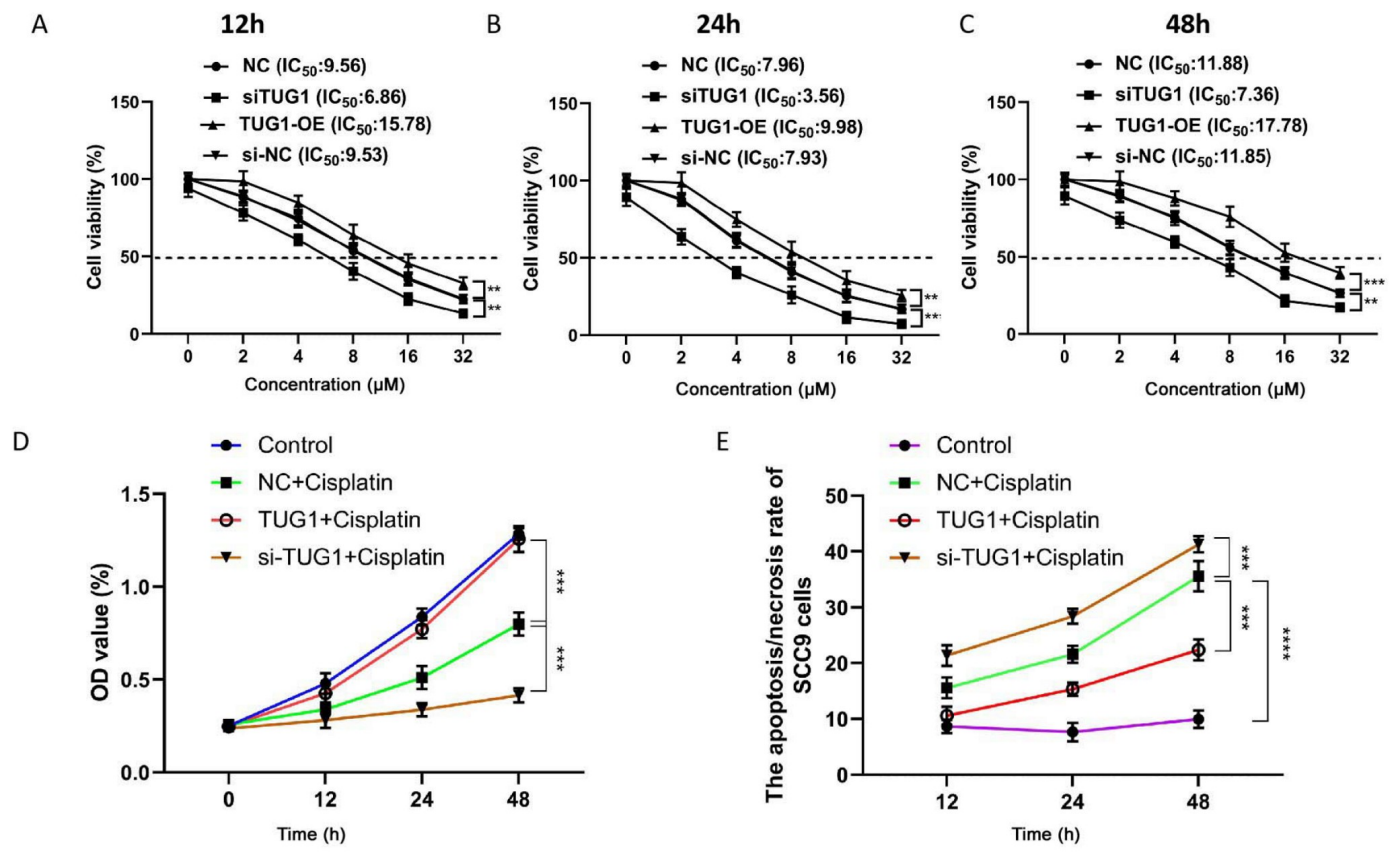
Figure 2 Viability and apoptosis of SCC9 cells after treatment with cisplatin (A) The cell viability of SCC‐9 cells was detected after 12 h of treatment with 2, 4, 8, 16, and 32 μM cisplatin. (B) Cell viability of SCC9 cells was detected after 24 h of treatment with 2, 4, 8, 16, and 32 μM cisplatin. (C) Cell viability of SCC9 cells was detected after 48 h of treatment with 2, 4, 8, 16, and 32 μM cisplatin. (D) The proliferation of SCC9 cells was detected in the control, si-TUG1+cisplatin, TUG1-OE+cisplatin, and NC+cisplatin groups after 12, 24, and 48 h of treatment with cisplatin. (E) SCC9 cell apoptosis was detected in the control, si-TUG1+cisplatin, TUG1-OE+cisplatin, and NC+cisplatin groups after 12, 24, and 48 h of treatment with cisplatin.

**Table:** **Table 1** Effect of cisplatin on cell viability

Cisplatin concentration	Host cell activity (OD _450nm_)	Survival rate (%)
‒	0.249±0.012	100
0.5% DMSO	0.246±0.003	‒
2 μg/mL	0.197±0.002	75.25
4 μg/mL	0.143±0.003	55.53
6 μg/mL	0.117±0.002	45.83
8 μg/mL	0.086±0.002	33.57

The DLX1, P53, and β-catenin are the key factors in cell apoptosis pathways, and overexpressions of DLX1, NF-κB, and β-catenin inhibit cell apoptosis and promote cell proliferation [8]. TNF-α and P53 promote cell apoptosis and inhibit cell proliferation [9]. Our results showed that DLX1, NF-κB, and β-catenin in the TUG1-OE group were significantly higher than those in the control group, and TNF-α and P53 expressions were significantly lower than those in the control group ( *P*<0.01). DLX1, NF-κB, and β-catenin in the si-TUG1 group were significantly lower than those in the si-NC group, while TNF-α and P53 were significantly higher than those in the si-NC group ( *P*<0.01) ( Supplementary Figure S1A,B). These results suggest that TUG1-OE promotes SCC9 cell proliferation and inhibits cell apoptosis. In contrast, si-TUG1 promotes SCC9 cell apoptosis and inhibits cell proliferation.

In summary, in the current study we found for the first time that lncRNA TUG1 is significantly overexpressed in SCC9 cells. High expression of lncRNA TUG1 could promote cell proliferation and cell migration, inhibit cell apoptosis, and promote malignant biological behaviors such as drug resistance, indicating that TUG1 may play an important role in the process of cell proliferation and apoptosis during the occurrence and development of OSCC. This study may provide a new idea for targeted therapy of OSCC.

## Supporting information

23059Supplementary_figure_S1

## Supplementary Data

Supplementary data is available at *Acta Biochimica et Biophysica Sinica* online.
