# Supplementary material for: Long noncoding RNA TUG1 promotes proliferation, migration and cisplatin resistance in oral squamous cell carcinoma: TUG1 promotes proliferation, migration and cisplatin resistance in OSCC
Source: Acta Biochim Biophys Sin (Shanghai). 2023 May 25;55(8):1323–6. doi: 10.3724/abbs.2023090 (PMC10448044; doi:10.3724/abbs.2023090)
Supplement: 23059Supplementary_figure_S1 [file 23059Supplementary_figure_S1.pdf]

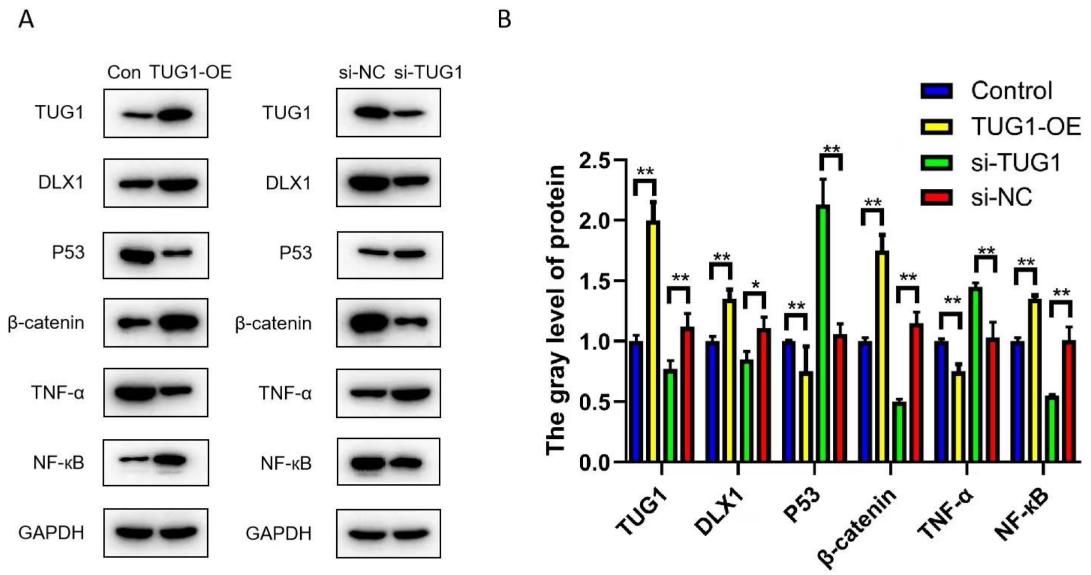


**Supplementary Figure S1. Effect of TUG1 on tumor-related genes in OSCC cells** (A) Western blots of DLX1, P53, NF-κB, β-catenin, MMP7, and TNF-α proteins after infection of OSCC cells transfected with TUG1-OE or TUG1 siRNA. (B) Quantitative analysis of DLX1, P53, NF-κB, β-catenin, MMP7, and TNF-α protein expressions in OSCC cells transfected with TUG1-OE or TUG1 siRNA.
